# Bringing the path toward an HIV-1 vaccine into focus

**DOI:** 10.1371/journal.ppat.1008663

**Published:** 2020-09-10

**Authors:** Cesar J. Lopez Angel, Georgia D. Tomaras

**Affiliations:** 1 Department of Microbiology and Immunology, Stanford University School of Medicine, Stanford, California, United States of America; 2 Duke Center for Human Systems Immunology, Duke University School of Medicine, Durham, North Carolina, United States of America; 3 Duke Human Vaccine Institute, Duke University School of Medicine, Durham, North Carolina, United States of America; 4 Department of Surgery, Duke University School of Medicine, Durham, North Carolina, United States of America; 5 Department of Pediatrics, Duke University School of Medicine, Durham, North Carolina, United States of America; 6 Department of Immunology, Duke University School of Medicine, Durham, North Carolina, United States of America; 7 Department of Molecular Genetics and Microbiology, Duke University School of Medicine, Durham, North Carolina, United States of America; University of Michigan Medical School, UNITED STATES

## Why do we need a human immunodeficiency virus (HIV-1) vaccine in the age of antiretroviral therapy and preexposure prophylaxis?

In the nearly four decades since its identification, HIV-1 has infected more than 70 million individuals worldwide, one-half of whom have succumbed to HIV-1-related illness [1]. Despite concerted preclinical studies and clinical trials by the scientific community during this time, the development of an HIV-1 vaccine remains elusive. Meanwhile, progress in antiretroviral therapy (ART) has rendered HIV-1 a chronic disease, with life expectancy approaching that of the general population when adequately treated [2]. These therapeutic advances led to “test and treat” guidelines to immediately start patients on ART upon diagnosis [3], with the goal of lowering viral loads to undetectable levels and effectively eliminating the risk of HIV-1 transmission to HIV-1–negative sexual partners [1, 4]. The “treatment as prevention” paradigm has resulted in approximately 80% of ART-treated individuals achieving undetectable viremia [1] and is now recommended for HIV-1–uninfected individuals at high risk of infection, in the form of daily prophylactic ART known as preexposure prophylaxis (PrEP). However, the efficacy of these interventions is pragmatically limited by the requirements for readily accessible HIV-1 testing, longitudinal clinical monitoring, availability of ART for the approximately 15 million individuals living with HIV-1 and not currently on treatment [1], and daily adherence to treatment for prolonged periods: a lack of which could potentially lead to drug-resistant strains. As a result, approximately 2 million individuals acquire HIV-1 globally every year despite our current prevention strategies [1]. Historically, vaccination represents the most cost-effective, scalable, and lasting public health intervention for the eradication of infectious disease; thus, developing a safe and effective HIV-1 vaccine is a global health imperative [5]. Importantly, an HIV-1 vaccine will be part of a multimodal array of HIV-1 prevention tools, and work on alternative preventive approaches should be extended and further developed until an effective vaccine becomes available.

## How close are we to an HIV-1 vaccine?

Most clinically approved vaccines confer immunity by inducing protective antibody responses. As the only viral determinants on the surface of HIV-1, the trimeric gp120 and gp41 HIV-1 envelope glycoproteins (Env), which mediate entry, are the primary targets of humoral immunity. Env trimers range from a metastable closed state to an open state when fully bound to CD4. Following CD4 binding, gp120 subunits undergo conformational changes that transiently expose coreceptor binding sites and lead to its dissociation from gp41. Subsequently, gp41 undergoes a step-wise transition that drives fusion of viral and target cell membranes. This metastability and conformational flexibility of Env, in conjunction with its tremendous genetic diversity and dynamic glycosylation states, allow HIV-1 to evade antibody neutralization and have frustrated vaccine development efforts.

To date, seven HIV-1 vaccine efficacy trials have been completed [6, 7]. The first two efficacy trials, VAX003 and VAX004, tested whether vaccine-induced antibodies against recombinant monomeric gp120 antigens could be protective or correlate with protection in injection drug users (VAX003) or in men who have sex with men (MSM) and women at high risk for infection (VAX004). Though these vaccines elicited high titers of anti-Env antibodies, they failed to induce antibodies capable of neutralizing a wide range of HIV-1 variants (i.e., broadly neutralizing antibodies [bNAbs]) or protect against HIV-1 acquisition. With greater appreciation for the role of T cells in controlling HIV-1, subsequent trials tested whether protection or reduced viral loads postinfection could be achieved by inducing anti–HIV-1 cellular immunity with vaccine formulations comprised of recombinant viral vectors encoding key HIV-1 antigens. The Step and closely related Phambili trials tested recombinant adenovirus serotype 5 (rAd5) vectors encoding HIV-1 Gag, Pol, and Nef proteins in MSM and women at high risk of infection (Step) or heterosexual men and women in South Africa (Phambili). The HIV Vaccine Trials Network 505 (HVTN 505) trial aimed to elicit both humoral and cellular responses by priming with DNA plasmids encoding *gag/pol/nef/env*, followed by a boost with rAd5 encoding a Gag-Pol fusion and Env proteins in men or transgender persons who have sex with men. These regimens showed no overall protection or reduction in viral load [8, 9], and a subset of vaccinees in the Step trial with preexisting immunity to Ad5 saw increased rates of HIV-1 infection [8]. Yet, Step also offered the first evidence that a viral vector vaccine could impose a selective immune pressure on transmitted virus [10]. The vaccine-induced sieve effect (determined by measuring genetic distance between transmitted and vaccine-encoded viral sequences) was observed in HIV-1 T cell epitopes encoded by the rAd5 vector in the Step trial [10]. The sieve effect in the HVTN 505 trial primarily focused on Env regions associated with infectivity, namely the CD4 binding site [11], and may have been mediated by humoral and/or cellular pressure [11–14].

In 2009, the RV144 “Thai trial” of a recombinant canarypox vector prime (ALVAC) and recombinant gp120 boost (AIDSVAX) tested in individuals at risk of heterosexual transmission became the first trial to show efficacy against HIV-1 infection by demonstrating 60.5% efficacy in the first year [6] that waned to 31.2% by three years postvaccination [15]. The rapid ebb of the immune response is an important shortcoming of this vaccination approach that has proven challenging to overcome. Nevertheless, models estimate that a vaccine with more than 50% efficacy for at least two years could significantly reduce the incidence of HIV-1 in high prevalence areas [5], so the modest efficacy of the immunization strategy used in the RV144 trial is an important benchmark that encourages cautious optimism that the path toward an HIV-1 vaccine is in view. The Uhambo trial tested in South Africa was a poxvirus prime, protein boost regimen like the RV144 vaccine regimen but with a different adjuvant, different envelope sequences [16, 17] and additional gp120 protein boosting [18, 19] with a goal of improving the breadth of immunity to subtype C and durability of vaccine-elicited antibody responses [20, 21]. The Uhambo trial was recently stopped after an interim analysis found that the regimen did not prevent HIV-1 infection [7], highlighting the need for systems immunology studies to explain the different results of the two trials and better understand the balance of immunity needed to achieve protection in different populations.

## What are the humoral correlates of protective HIV-1 vaccination?

Vaccine-induced humoral responses are broadly binned into neutralizing antibody functions mediated by the antibody antigen-binding fragment (F_ab_) and antibody effector functions mediated by an antibody’s fragment crystallizable (F_c_) region engaging its receptor (FcR) on innate immune cells, i.e., antibody-dependent cellular cytotoxicity (ADCC), antibody dependent cellular phagocytosis (ADCP), complement activation, and innate immune cell activation. Most chronically infected individuals generate antibody responses with a modest degree of cross-neutralization breadth and potency. After years of sustained viremia, 10% to 20% of these individuals develop highly potent bNAbs that, despite their broad neutralization activity, are incapable of controlling the host’s infection due to viral Env evolution outpacing the adaptive response. Production of these naturally occurring bNAbs is influenced by transmitted viral antigens [22] and is due to extensive somatic hypermutation of rare, low affinity, naïve B cell receptors (BCRs). Notably, BCRs that ultimately result in bNAbs exhibit atypical structural and binding characteristics that are frequently negatively selected during B cell development (reviewed in [23]). Immunogens tested in vaccine candidates to date have failed to overcome these challenges to elicit bNAbs, but the protective potential of bNAbs has been repeatedly demonstrated by passive immunization and challenge of nonhuman primates (NHPs) (reviewed [23]). The ongoing Antibody Mediated Prevention (AMP) trial is the first efficacy study testing passive immunization in humans [24] and will provide the first insight as to whether immunoprophylaxis of a single bNAb targeting the CD4bs region of the HIV-1 envelope can also protect humans. The results from the AMP trial will inform future plans to test combinations of different bNAbs designed to have improved potency and breadth against circulating viruses.

In contrast, NHP protection studies [23, 25] and clinical trials have underscored the important role of antibodies with F_c_ effector functions in protective vaccination. Case-control analysis of the RV144 trial vaccines revealed that the magnitude of immunoglobulin G (IgG) antibodies targeting Env variable regions 1 and 2 (V1V2), correlated with decreased HIV-1 risk, while the titer of specific serum immunoglobulin A (IgA) antibodies correlated with increased risk of infection [26]. Moreover, antibodies elicited in the RV144 trial were polyfunctional and promoted ADCC, ADCP, and complement activation [23, 27]. Similar analyses of the HVTN 505 trial also revealed strong inverse correlations between HIV-1 acquisition and Env-IgG binding [12] and Env IgG3 breadth, FcγRIIa binding, and ADCP [14]. In both trials, the efficacy of the vaccine was modified by the presence of single nucleotide polymorphisms at the FcR locus, further supporting FcR-mediated protective mechanisms [14, 28].

## Are there cellular immune correlates of protective HIV-1 vaccination?

With the development of a new computational tool for combinatorial polyfunctionality analysis of antigen-specific T-cell subsets (COMPASS), an Env-specific polyfunctional CD4^+^ T cell signature was identified in the RV144 trial that correlated with decreased HIV-1 risk and matched the significance of the primary V1V2 IgG correlate of decreased HIV-1 risk in that trial [29]. Considering that the development and maturation of the optimal antibody responses associated with protective vaccination described above require help from CD4^+^ T cells, this finding suggests that a coordinated humoral and cellular response may be necessary for protection.

Conversely, while CD8^+^ T cells effect many antiviral functions, the CD8^+^ T cell responses in the RV144 trial were limited [15]. In the HVTN 505 trial, however, COMPASS helped identify a strong inverse correlation between HIV-1 acquisition and the abundance and polyfunctionality of Env-specific CD8^+^ T cells [13]. These findings highlight the importance of sophisticated analytical approaches to dissect complex single-cell data to aid the search for cellular immune correlates of protective vaccination.

## What does the path to an HIV-1 vaccine look like from here?

**Fig 1:**
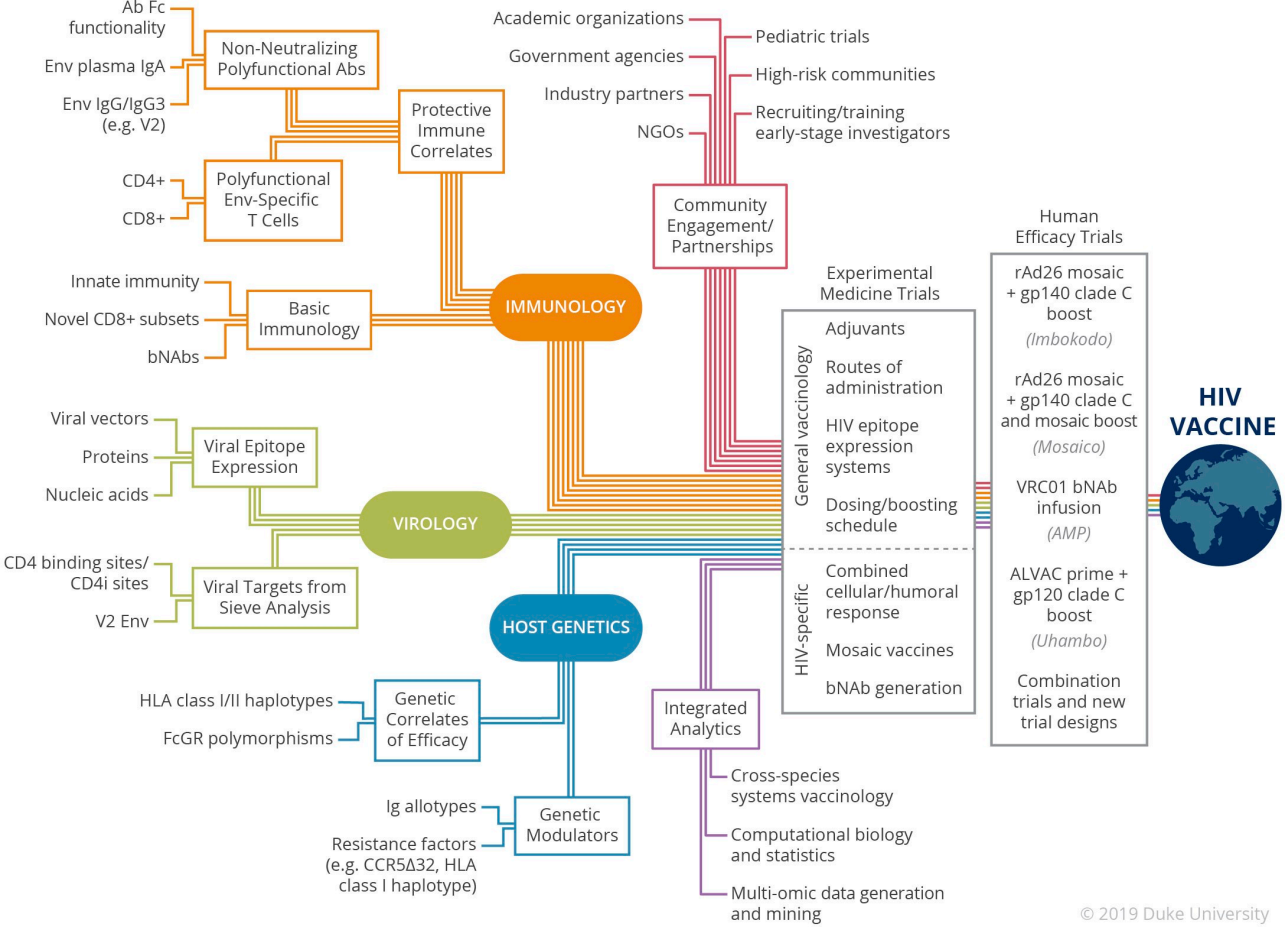
Focusing diverse efforts to achieve an HIV-1 vaccine. The development of a safe and effective HIV-1 vaccine is a confluence of scientific efforts in immunology (orange), virology (green), and genetics (blue), is boosted by advanced analytical pipelines (purple), and is inextricably linked to community implementation and reliant on partnerships with key stakeholders (red). Knowledge of the protective immune and genetic correlates and crucial viral targets yielded from previous efficacy trials is combined with basic concepts in immunology, viral epitope expression, and genetic modulators of HIV-1 acquisition to test novel vaccines in clinical and experimental medicine trials. Analyses of the immune responses elicited in the global clinical efficacy trials that are ongoing, and recently completed (Uhambo), will inform our understanding of the balance of immunity that favors long-lasting protective immunity. Looking forward, there is a robust pipeline of innovative immunogen and trial design strategies to build upon the knowledge gained from the outcomes of these trials.

The HIV-1 vaccine field sits at a nexus where immunology, virology, genetics, translational medicine, computational analytics, and community engagement converge (Fig 1). Ongoing vaccine trials are guided by the immune correlates of protection defined above and include combination efficacy studies that incorporate PrEP alongside vaccine regimens (PrEPVacc) and tests of new viral vectors. Leading vector candidates include modified vaccinia Ankara (MVA) [6, 21], cytomegalovirus (CMV) vectors that induce persistent novel CD8^+^ T cell responses [30], and rAd26 vectors that differ from previous rAd5 vectors in part by lower preexisting antivector neutralizing antibody titers in human populations [23]. The ongoing Imbokodo (HVTN 705/HPX2008; NCT03060629) and Mosaico (HVTN 706/HPX3002/Mosaico; NCT03964415) efficacy trials utilize rAd26 vectors encoding epitope sequences computationally optimized to capture the diversity of multiple global HIV-1 strains, i.e., mosaic immunogens, followed by Env protein boosting [18].

Vaccine immunogen design strategies have increasingly focused on novel methods to systematically train the immune system to elicit bNAbs, such as shepherding the production of bNAbs from germline BCRs, antibody lineage-based immunogen designs, strategies to elicit multi-epitope bNAb responses, and epitope focusing strategies [24, 31]. These efforts have been enhanced by SOSIP immunogens: stabilized Env trimers engineered to mimic the native virion trimer [24], with the goal of eliciting more physiologically relevant, potent bNAbs, in comparison to gp120 monomers. While SOSIPs have yielded some success in animal models [24, 32], the immunological differences across species compel proof of concept experimental medicine trials to directly test the capacity of the human immune system to be “trained” to elicit protective immunity. To that end, a leading SOSIP candidate, SOSIP.664 based on HIV-1 strain BG505, advanced to human trials in 2018 [24].

A major challenge for HIV-1 vaccinologists has been incorporating the interconnectivity and heterogeneity of the human immune system into evaluations of vaccine efficacy and the search for correlates of protection. Novel analytical tools have bolstered this search by dissecting heterogenous host genetics and vaccination responses to identify characteristics predictive of outcome. Such systems vaccinology approaches integrate systems biology methodologies into the historically empirical field of vaccinology and have already identified genetic correlates of HIV-1 vaccine efficacy (e.g., HLA haplotypes [6, 33] and FcR polymorphisms [6, 14, 28, 34]) and genetic modulators of HIV-1 vaccination responses, e.g., Ig allotypes [35]. Analyses at the intersection of immunology, host genetics, and viral genetics will likely uncover new insights into human immunity that could be leveraged for vaccine development more broadly and enable vaccinologists to precisely guide the administration of these vaccines to individuals most likely to benefit.

Indeed, identifying communities most likely to benefit from an HIV-1 vaccine, such as populations with high prevalence of HIV-1 and *Mycobacterium tuberculosi*s coinfection, is important when designing and recruiting for future trials. Similarly, a proven efficacious vaccine that can be safely administered early in life may offer the best opportunity for prevention [36]. Integrative data analysis from knowledge gained from the intersections of immunology, virology, and host genetics, along with an informed understanding of implementation strategies tuned specifically for optimal effectiveness in the affected communities [37], is on the horizon.
